# Analysis of Vaginal Microbiota Variations in the Third Trimester of Pregnancy and Their Correlation with Preterm Birth: A Case-Control Study

**DOI:** 10.3390/microorganisms12020417

**Published:** 2024-02-19

**Authors:** Catalin Prodan-Barbulescu, Felix Bratosin, Roxana Folescu, Estera Boeriu, Zoran Laurentiu Popa, Cosmin Citu, Adrian Ratiu, Ovidiu Rosca, Adrian Cosmin Ilie

**Affiliations:** 1 Doctoral School, “Victor Babes” University of Medicine and Pharmacy Timisoara, Eftimie Murgu Square 2, 300041 Timisoara, Romania; catalin.prodan-barbulescu@umft.ro; 2 IInd Surgery Clinic, “Victor Babes” University of Medicine and Pharmacy Timisoara, Eftimie Murgu Square 2, 300041 Timisoara, Romania; 3 Department I, Discipline of Anatomy and Embriology, “Victor Babes” University of Medicine and Pharmacy Timisoara, Eftimie Murgu Square 2, 300041 Timisoara, Romania; 4 Department of Infectious Diseases, “Victor Babes” University of Medicine and Pharmacy Timisoara, Eftimie Murgu Square 2, 300041 Timisoara, Romania; ovidiu.rosca@umft.ro; 5 Methodological and Infectious Diseases Research Center, Department of Infectious Diseases, “Victor Babes” University of Medicine and Pharmacy Timisoara, Eftimie Murgu Square 2, 300041 Timisoara, Romania; 6 Department of Family Medicine, “Victor Babes” University of Medicine and Pharmacy Timisoara, Eftimie Murgu Square 2, 300041 Timisoara, Romania; folescu.roxana@umft.ro; 7 Department of Pediatrics, “Victor Babes” University of Medicine and Pharmacy Timisoara, Eftimie Murgu Square 2, 300041 Timisoara, Romania; estera.boeriu@umft.ro; 8 Department of Obstetrics and Gynecology, “Victor Babes” University of Medicine and Pharmacy Timisoara, Eftimie Murgu Square 2, 300041 Timisoara, Romania; popa.zoran@umft.ro (Z.L.P.); citu.ioan@umft.ro (C.C.); ratiu.adrian@umft.ro (A.R.); 9 Department III Functional Sciences, Division of Public Health and Management, “Victor Babes” University of Medicine and Pharmacy Timisoara, 300041 Timisoara, Romania; ilie.adrian@umft.ro

**Keywords:** vaginal microbiota, preterm birth, obstetric outcomes

## Abstract

This study conducted a detailed analysis of the vaginal microbiota in pregnant women to explore its correlation with preterm birth (PTB) outcomes. The primary objective was to identify microbial variations associated with increased PTB risk. Secondary objectives included investigating how changes in microbial composition relate to the local immune environment and PTB. Utilizing a retrospective case–control design, the study involved pregnant women with liveborn infants between 2019 and 2023. In total, 89 women who delivered preterm and 106 term deliveries were included. Data collection focused on third-trimester vaginal cultures. Statistically significant differences were observed between the preterm and full-term groups in several areas. The median white blood cell count (10.2 × 10^3^/mm^3^ vs. 7.6 × 10^3^/mm^3^, *p* = 0.009) and neutrophil count (7.2 × 10^3^/mm^3^ vs. 5.1 × 10^3^/mm^3^, *p* < 0.001) were higher in the preterm group. Vaginal pH was also elevated in preterm births (5.6 vs. 4.4, *p* < 0.001), with a higher prevalence of bacterial vaginosis (29.2% vs. 12.3%, *p* = 0.001) as indicated by the Nugent Score. The study noted a significant association of PTB with the presence of *Candida* spp. (OR = 1.84, *p* = 0.018), *Gardnerella vaginalis* (OR = 2.29, *p* = 0.003), *Mycoplasma hominis* (OR = 1.97, *p* = 0.007), and *Ureaplasma urealyticum* (OR = 2.43, *p* = 0.001). Conversely, a reduction in *Lactobacillus* spp. correlated with a decreased PTB risk (OR = 0.46, *p* = 0.001). The study provides compelling evidence that specific vaginal microbiota components, particularly certain pathogenic bacteria and an altered Lactobacillus profile, are significantly associated with PTB risk. These findings highlight the potential of targeting microbial factors in strategies aimed at reducing PTB rates. Further research is necessary to fully understand the complex interplay between microbial dynamics, host immunity, and PTB outcomes.

## 1. Introduction

Preterm birth, defined as delivery before 37 weeks of gestation, remains a leading cause of neonatal morbidity and mortality worldwide [1,2]. Despite advances in neonatal care, the rates of preterm births have not significantly decreased worldwide in recent years, highlighting the need for a deeper understanding of its etiology [3,4]. Emerging research has increasingly pointed to the role of the vaginal microbiota in maternal and neonatal health, suggesting a significant association between microbial dysbiosis and adverse pregnancy outcomes, including preterm birth [5,6]. The vaginal microbiome, a complex ecosystem of bacteria, varies widely among women and changes throughout pregnancy, influenced by factors such as genetics, environment, and lifestyle [7,8].

Recent studies have delineated a more detailed landscape of the vaginal microbiota, identifying specific bacterial genera and species that predominate in healthy pregnancies versus those associated with negative pregnancy outcomes [9,10]. *Lactobacillus* species, for example, are typically dominant in a healthy vaginal microbiome and are known for their protective roles, producing lactic acid and bacteriocins that inhibit the growth of pathogenic organisms [11]. Conversely, an increase in microbial diversity and the presence of certain anaerobic bacteria like *Gardnerella* and *Ureaplasma* have been linked to inflammation, infection, and, subsequently, an increased risk of birth risks [12,13].

The immunological interactions between the vaginal microbiota and the host are crucial in maintaining pregnancy. A healthy microbiome contributes to the establishment of a tolerant immunological environment necessary for the successful continuation of pregnancy. However, dysbiosis can lead to an imbalance in pro-inflammatory and anti-inflammatory responses, potentially triggering pathways that lead to negative pregnancy outcomes [14,15]. Inflammation, particularly in the context of infection, is a well-recognized pathogenic pathway in preterm birth, and microbiota alterations are a key component in this process.

Technological advances in metagenomics and bioinformatics have allowed for more comprehensive and accurate profiling of the vaginal microbiome, providing insights into its structure, function, and dynamics during pregnancy [16,17]. These tools have facilitated large-scale studies that correlate specific microbiota profiles with pregnancy outcomes. However, there remains a gap in understanding the precise mechanisms by which these microbial communities influence the risk of preterm birth and how interventions can be tailored to modulate the microbiome to prevent negative outcomes.

This study aims to address these gaps by conducting a detailed analysis of the vaginal microbiota in a cohort of pregnant women and correlating these findings with preterm birth outcomes. The primary hypothesis is that specific variations in the vaginal microbiota are associated with an increased risk of preterm birth. Secondary hypotheses include the supposition that alterations in the microbial composition are linked to changes in the local immune environment, which in turn contribute to the risk of preterm delivery. The objectives of this study are to identify specific microbial species associated with preterm birth, understand the temporal dynamics of the vaginal microbiota throughout pregnancy, and explore the potential differences and microbial dysbiosis between pregnant women who gave birth preterm and full-term. 

## 2. Materials and Methods

### 2.1. Study Design

The current study followed a case–control design, with data collected from pregnant patients from the Obstetrics and Gynecology unit of the Clinical County Hospital admitted between 2019 and 2023. The hospital’s Institutional Review Board granted ethical approval, adhering to the Declaration of Helsinki’s guidelines, which was approved on 31 March 2023 (code E-1853). Data were retrospectively assessed, and all participants provided informed consent for personal and medical records being used in research studies at the time of evaluation. Confidentiality and privacy of patient data were strictly maintained.

Information regarding the patients’ health was collected from their clinical paper records and electronic medical records. Vaginal swabs were taken to analyze the microbiota and check for antimicrobial resistance during hospital admission or appointments made in private clinics in order to determine asymptomatic high-risk pregnancy infections and changes in the vaginal microbiota, especially reductions in lactobacilli and the emergence of bacteria resistant to treatment. All patients were sampled in the third trimester of pregnancy and bacterial cultures were performed.

### 2.2. Selection Criteria

The group of cases comprised patients who gave birth preterm, while the control group included those who gave birth at term. The inclusion criteria comprised the following: (1) pregnant women aged 18 years and above; (2) complete medical records available for the study period; (3) consent to allow data collection; (4) vaginal cultures performed during the third trimester of pregnancy.

Patients were excluded if they presented with obstetrical complications (such as the preterm premature rupture of membranes, pre-eclampsia, or cervical insufficiency), multifetal pregnancy, fetal growth restriction, placental abnormalities (placenta previa, placental abruption, placenta accreta spectrum), gestational hypertension, or gestational diabetes. Patients with incomplete or missing records were not considered for analysis. 

Patients with a history of chronic diseases that could independently affect the vaginal microbiome or pregnancy outcomes, such as autoimmune diseases, were excluded. Those who received antibiotic treatment within two weeks prior to sampling were also excluded to avoid the influence of antibiotics on the microbial analysis. Moreover, those having used antenatal corticosteroids were also excluded. Furthermore, patients with a known history of substance abuse or who had undergone any cervical procedures during pregnancy, which could affect the microbiota or pregnancy outcomes, were not included in this study. Sexually transmitted infections, including *Chlamydia trachomatis*, *Neisseria gonorrhoeae*, and *Trichomonas vaginalis*, were also excluded, as these are known risk factors for negative birth outcomes [18].

### 2.3. Study Variables

The study variables included demographic information, lifestyle factors (alcohol and smoking), medical history, and laboratory results. Demographics included age and Body Mass Index (BMI), while medical history included data regarding patients’ parity, previous UTI, hypertension, diabetes, anemia, respiratory infections during pregnancy, and diarrheal illness during pregnancy. Laboratory data comprised the following blood tests: WBC, lymphocytes, neutrophils, PLT, RBC, hemoglobin, CRP, creatinine, and urea. Newborn features included gestational weight, gestational age, and type of birth (vaginal, cesarean, or assisted birth). Microbial identification focused on Gram-staining vaginal smears, vaginal culture results, and vaginal pH. 

Intermediate microbiota was characterized by the presence of streaks of bacterial vaginosis microbiota in a smear that also displayed areas of normal or differing microbiota [19]. Full bacterial vaginosis was identified by a complete lack of lactobacilli, typical granular microflora with innumerable bacteria, and the presence of clue cells. Coccoid aerobic vaginitis microbiota was another category, distinguished by its own unique characteristics. Lastly, primarily staphoid aerobic vaginitis microbiota, where a Candida blastospore was visible, was noted for its similarities to the microbiota observed in intermediate microbiota, especially in the upper left corner of the smear, and the striking resemblances it shared with both the coccoid and staphoid aerobic vaginitis microbiota.

### 2.4. Statistical Analysis

Data analysis was performed using SPSS version 27. A convenience method was considered to calculate the sample size, using a confidence level of 95%, for a 5% margin of error and a 10% proportion of preterm births in the general population worldwide [20], with a necessary minimum of 139 cases. Descriptive statistics provided a summary of demographic and clinical characteristics. The proportions of microbial species and resistance patterns between the two groups were compared using the Chi-square test or the Fisher’s exact test, based on the frequency assumptions. For continuous data, we calculated the independent samples *t*-test when comparing two means, and the Mann–Whitney u-test to compare two medians. Pearson’s correlation coefficient was calculated for continuous variables, while Spearman’s rho was computed for categorical data. Logistic regression models were used to identify preterm birth risk factors independently associated with microbial complexity and resistance patterns. The analysis was adjusted for the confounding factors, including gestational age, BMI, smoking status, and parity, to ensure the robustness of findings. We calculated the False Discovery Rate using the Benjamin–Hochberg method. A *p*-value < 0.05 was considered statistically significant.

## 3. Results

A total of 89 pregnant women who gave birth preterm were included in the study, and 106 who gave birth full-term, respectively. Table 1 presents the demographic and health characteristics of the study participants. The mean age of participants in the preterm group was 28.6 years, compared to the full-term group’s mean age of 29.1 years, although this difference was not statistically significant (*p* = 0.502). The age categories further detailed that 69.7% of the preterm group and 74.5% of the full-term group were under 35 years of age, with the remaining participants being 35 years or older (*p* = 0.449). The percentage of participants with a BMI of 30 kg/m^2^ or greater was slightly higher in the preterm group (19.1%) compared to the full-term group (15.1%), but this difference was not statistically significant (*p* = 0.457).

Lifestyle factors such as smoking and alcohol use during pregnancy were examined. In the preterm group, 13.5% reported smoking during pregnancy compared to 7.5% in the full-term group, and 5.6% of the preterm group reported alcohol use during pregnancy compared to 0.9% in the full-term group. Parity did not show a significant difference between the groups, with 59.6% primigravida and 40.4% multigravida in the preterm group compared to 59.4% and 40.6%, respectively, in the full-term group (*p* = 0.986). Medical history variables such as Urinary Tract Infections (UTIs), hypertension, diabetes, anemia, respiratory infections, and diarrheal illness during pregnancy were also compared. The preterm group had a higher percentage of UTIs (24.7% vs. 14.2%), but this difference was marginally not significant (*p* = 0.060). Hypertension, diabetes, anemia, respiratory infections, and diarrheal illness showed no statistically significant differences between the groups.

The white blood cell count showed a statistically significant difference between the two groups. The median WBC count was higher in the preterm group (10.2 × 10^3^/mm^3^) compared to the full-term group (7.6 × 10^3^/mm^3^) with a *p*-value of 0.009, suggesting a possible association between elevated WBC counts and the incidence of preterm births. Neutrophil counts were also significantly different between the groups. Preterm births were associated with a higher median neutrophil count (7.2 × 10^3^/mm^3^) compared to full-term births (5.1 × 10^3^/mm^3^) with a *p*-value of less than 0.001.

However, no statistically significant differences were observed in the lymphocyte counts, platelet (PLT) counts, red blood cell (RBC) counts, hemoglobin levels, C-Reactive Protein (CRP) levels, creatinine, or urea between the preterm and full-term groups. The *p*-values for these tests ranged from 0.109 to 0.753, indicating no strong evidence of difference in these parameters between the two groups, as presented in Table 2.

The gestational weight of the newborns was significantly different between the two groups (*p* < 0.001). In the preterm birth group, fewer babies weighed over 2500 g (52.8%) compared to the full-term birth group (84.9%). Conversely, higher percentages of lower-weight categories were observed in the preterm group: 3.4% weighed 500–999 g, 9.0% weighed 1000–1499 g, and 34.8% weighed 1500–2499 g. In contrast, in the full-term group, only 0.9% weighed 1000–1499 g, and 14.2% weighed 1500–2499 g. This significant difference in weight distribution underscores the association between lower birth weight and preterm deliveries.

Regarding gestational age, all preterm births occurred before 37 weeks, with 7.9% being early preterm (<28 weeks), 21.3% moderate preterm (28–32 weeks), and 70.8% later preterm (32–36 weeks). In contrast, the full-term births included early-term (17.0%), full-term (69.8%), and post-term (13.2%) babies, highlighting the clear demarcation in gestational age between the preterm and full-term groups.

The type of birth also showed a significant difference (*p* < 0.001) between the two groups. The preterm group had a higher percentage of cesarean deliveries (65.2%) compared to the full-term group (18.9%). Vaginal deliveries were more common in the full-term group (73.6%) than in the preterm group (28.1%). Assisted deliveries were relatively low in both groups but were slightly more common in the full-term group (7.5%) compared to the preterm group (6.7%), as seen in Table 3.

The pH test results indicated a higher median pH value for the preterm group (5.6) compared to the full-term group (4.4), with a highly significant *p*-value of less than 0.001. The overall assessment based on the Nugent Score, which categorizes the vaginal microbiota as normal, intermediate, or showing bacterial vaginosis, showed significant differences between the two groups (*p* = 0.001). In the preterm group, 39.3% had normal microbiota compared to 64.2% in the full-term group, indicating a higher prevalence of normal microbiota in full-term births. Intermediate microbiota was found in 31.5% of the preterm group and 23.6% of the full-term group. Notably, a higher percentage of the preterm group (29.2%) had bacterial vaginosis compared to the full-term group (12.3%), suggesting that bacterial vaginosis is more prevalent among women who deliver preterm.

**Figure 1:**
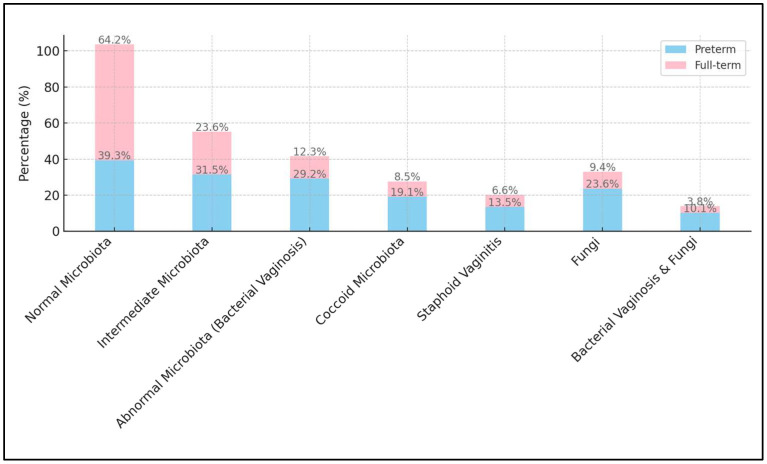
Vaginal smear results by preterm and full-term births.

In examining particular microbiota types, coccoid microbiota was more common in the preterm group (19.1%) compared to the full-term group (8.5%), with a *p*-value of 0.029, indicating a possible association with preterm birth. Staphoid vaginitis was also more prevalent in the preterm group (13.5% vs. 6.6%); however, the difference was not statistically significant (*p* = 0.106). Fungal infection, indicated by the presence of fungi, was significantly more common in the preterm group (23.6% vs. 9.4%, *p* = 0.007). The combination of bacterial vaginosis and fungi was also more frequent among the preterm group (10.1% vs. 3.8%), although this result was marginally not significant (*p* = 0.077), as presented in Table 4 and Figure 1.

The presence of *Candida* spp. was significantly higher in the preterm group (24.7%) compared to the full-term group (9.4%), with a *p*-value of 0.004. Similarly, the prevalence of *Gardnerella vaginalis* was considerably higher in the preterm group (25.8%) than in the full-term group (12.3%), with a *p*-value of 0.015. A significant reduction in *Lactobacillus* spp. was noted among the preterm group (73.0%) compared to the full-term group (87.7%), with a *p*-value of 0.009. *Mycoplasma hominis* was also significantly more prevalent in the preterm group (16.9%) compared to the full-term group (5.7%), with a *p*-value of 0.012. *Ureaplasma urealyticum* was found more frequently in the preterm group (14.6%) than in the full-term group (3.8%), with a significant *p*-value of 0.007.

Non-significant findings, including the presence of *Actinomyces* spp., *Bacillus* spp., *Corynebacterium* spp., *Enterococcus* spp., *Escherichia coli*, *Haemophilus influenzae*, *Klebsiella* spp., *Peptostreptococcus anaerobius*, *Staphylococcus aureus*, *Staphylococcus epidermidis*, *Staphylococcus haemolyticus*, *Streptococcus agalactiae*, *Streptococcus anginosus*, *Streptococcus mitis*, and *Streptococcus salivarius*, did not show a strong correlation with preterm or full-term births, as presented in Table 5.

A significant negative correlation was found between gestational age and white blood cell (WBC) count (rho = −0.307 *), indicating that as gestational age decreases, indicative of preterm birth, WBC counts tend to increase. Vaginal pH showed a strong negative correlation with gestational age (rho = −0.452 *), suggesting that lower gestational ages are associated with higher vaginal pH levels.

*Lactobacillus* spp. had a positive correlation with gestational age (rho = 0.406 *) and a strong negative correlation with vaginal pH (rho = −0.559 *). *Candida* spp. showed a positive correlation with vaginal pH (rho = 0.308 *), implying that yeast infections are associated with higher pH levels, which could contribute to adverse pregnancy outcomes. Additionally, there was a negative correlation between *Candida* spp. and *Lactobacillus* spp. (rho = −0.256 *), highlighting the competitive relationship between these microorganisms.

*Mycoplasma hominis* exhibited negative correlations with gestational age (rho = −0.217 *) and *Lactobacillus* spp. (rho = −0.312 *) and positive correlations with vaginal pH (rho = 0.357 *) and *Ureaplasma urealyticum* (rho = 0.504 *), suggesting its association with conditions leading to preterm birth. *Ureaplasma urealyticum* correlated negatively with gestational age (rho = −0.259 *) and *Lactobacillus* spp. (rho = −0.359 *) and positively with vaginal pH (rho = 0.418 *), indicating its role in the microbial imbalance associated with preterm birth.

**Figure 2:**
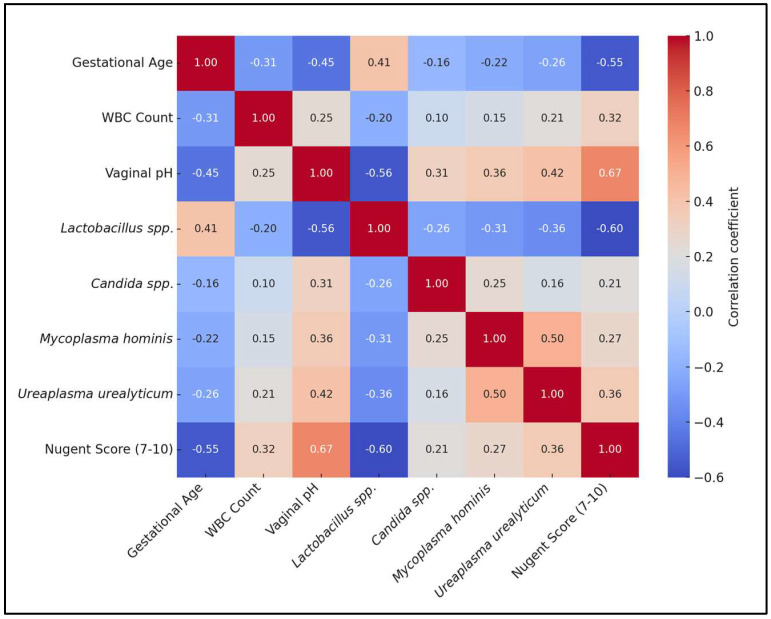
Correlation matrix heatmap.

The Nugent Score, which is used to diagnose bacterial vaginosis, showed the strongest negative correlation with gestational age (rho = −0.551 *), indicating that higher scores (and thus more severe bacterial vaginosis) are strongly associated with preterm births. It also had a substantial positive correlation with vaginal pH (rho = 0.672 *) and a negative correlation with *Lactobacillus* spp. (rho = −0.601 *), reinforcing the link between bacterial vaginosis, pH imbalance, and decreased beneficial bacteria, as presented in Table 6 and Figure 2.

The presence of *Candida* spp. in vaginal cultures was found to increase the odds of preterm birth by 84% (OR = 1.84, *p* = 0.018), while the presence of *Gardenerella vaginalis* more than doubled the risk (OR = 2.29, *p* = 0.003). *Mycoplasma hominis* and *Ureaplasma urealyticum* were also identified as significant contributors to the likelihood of preterm delivery, with odds ratios of 1.97 (*p* = 0.007) and 2.43 (*p* = 0.001), respectively, indicating a near doubling of risk with their presence. Notably, an increased vaginal pH greater than 4.5 was associated with a 68% increase in preterm birth risk (OR = 1.68, *p* = 0.014), and a high Nugent Score (7–10), indicative of bacterial vaginosis, was strongly linked to preterm birth with an odds ratio of 2.83 (*p* < 0.001).

**Figure 3:**
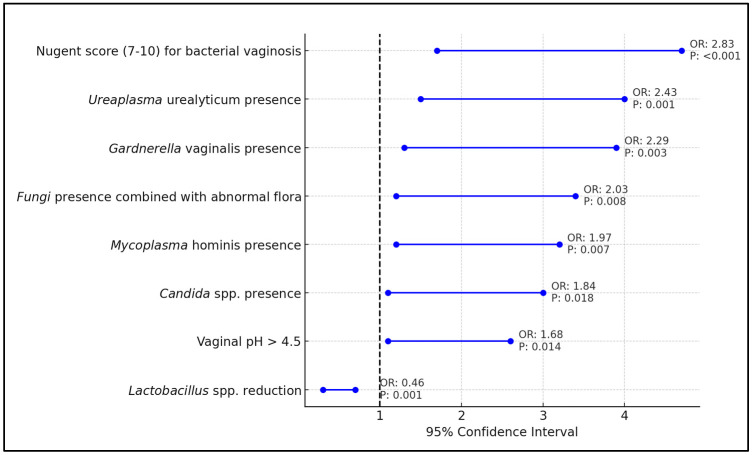
Forest plot of significant risk factors associated with preterm birth.

Conversely, a reduction in *Lactobacillus* spp., typically considered protective against pathogenic invasion and imbalance, was associated with a significantly decreased risk of preterm birth (OR = 0.46, *p* = 0.001), underscoring its importance in vaginal health. The combination of fungi presence with abnormal microbiota further increased the risk of preterm delivery (OR = 2.03, *p* = 0.008), suggesting a complex interplay of microbial factors contributing to adverse pregnancy outcomes, as presented in Table 7 and Figure 3.

## 4. Discussion

### 4.1. Literature Findings

This study found important correlations between the vaginal microbiome and preterm birth, shedding light on the potential pathways through which infections and microbial imbalances can lead to early labor. The significant increase in white blood cells and neutrophil counts in preterm births compared to full-term births is a notable finding, indicating an inflammatory response that may be triggered by infection or other stressors [21,22]. The specificity of this inflammatory response, as suggested by the lack of significant differences in other markers like lymphocytes and CRP, points to a potentially localized immune reaction within the reproductive tract. Understanding these inflammatory pathways is crucial for developing targeted interventions to prevent or mitigate preterm labor.

The analysis of vaginal microbiota revealed a distinct microbial environment in preterm births. The higher prevalence of bacterial vaginosis and the significant increase in vaginal pH in the preterm group indicate a shift towards an unfavorable microbial microbiota [23,24]. Bacterial vaginosis is characterized by a reduction in protective *Lactobacillus* species and an increase in diverse anaerobic bacteria, which can lead to inflammation and increased susceptibility to infections, both of which are risk factors for preterm labor. The presence of specific microorganisms, like *Candida* spp., *Gardnerella vaginalis*, *Mycoplasma hominis*, and *Ureaplasma urealyticum*, further supports the notion that a disturbed vaginal microbiome is associated with adverse pregnancy outcomes. These organisms have been linked to inflammation, ascending infections, and other mechanisms that could compromise the uterine environment and lead to preterm birth.

The correlations between microbial presence, vaginal pH, and Nugent Scores with gestational age highlight the intricate relationship between the host and its microbiome. For instance, the negative correlation between *Lactobacillus* spp. and vaginal pH suggests that as the beneficial lactobacilli decrease, the vaginal environment becomes more alkaline, which is conducive to the growth of harmful bacteria. Similarly, the positive correlation of pathogenic bacteria with Nugent Scores and pH indicates their association with a more hostile vaginal environment. These findings underscore the potential for using the vaginal microbiome’s composition as a predictive tool for preterm birth and as a target for therapeutic interventions.

Similarly, the study by Liu et al. revealed significant differences in the abundance and diversity of vaginal microbiota among women who gave birth preterm, although the researchers utilized more advanced methods such as metagenomic analysis, where the alpha diversity in participants with preterm rupture of membranes (PROM) was significantly lower compared to the that in the preterm group [25]. *Lactobacillus* spp. was predominantly abundant in the preterm birth group, as determined by the Linear discriminant analysis effect size. Another study also noted differences in vaginal microbiota composition across different ethnicities, with Asian and Caucasian pregnant women exhibiting the lowest alpha diversity [26]. Research from South Korea and London using a 16S metagenomics approach indicated that pregnant women at risk for preterm prelabor rupture of membranes (PPROM) showed greater abundance and diversity upon admission compared to those prone to preterm birth without PROM, highlighting the significance of vaginal microbiota in PROM outcomes [27].

Parallel to these findings, Romero et al. [28] found no difference in vaginal microbiota abundance between full-term and spontaneous preterm women. However, Caucasian mothers experiencing preterm birth exhibited significantly lower alpha diversity compared to full-term mothers [26], while African-American mothers showed higher diversity when delivering preterm [29].

It is also essential to emphasize the importance of *Lactobacillus* spp. in the vaginal microbiome, acting as a barrier and indicating reproductive health, as suggested by previous research [30]. Therefore, pregnant women with a high abundance of *Gardnerella* and a low abundance of *Lactobacillus* spp. were found to have a higher risk of preterm birth [31]. Interestingly, the association varied based on gestational age during sample collection; after 12 weeks, women with preterm birth had lower alpha diversity, but this trend reversed for samples taken before 12 weeks [32]. Despite these findings, some studies reported a weak or non-existent association between *Lactobacillus*-dominant communities and preterm birth [33].

In their study, Brown et al. observed that women at risk for preterm birth exhibited the highest decrease in *Lactobacillus* spp. between 24 and 29 weeks of gestation, accompanied by an enrichment of opportunistic bacteria such as *Prevotella*, *Streptococcus*, *Peptoniphilus*, *Ureaplasma*, and *Dialister* spp., which are known to upregulate metalloproteinases and pro-inflammatory cytokines while diminishing the effects of tissue inhibitor metalloproteinases [34,35]. In contrast, women with term deliveries experienced reduced microbial richness as gestation progressed, with *Lactobacillus* spp. dominating post 24 weeks. This pattern aligns with a meta-analysis that described a crucial “immune clock” phase of pregnancy, indicating an increased sensitivity to pathogenic vaginal bacteria during 20–30 weeks of gestation [36].

Although our study analyzed a homogenous population of Caucasian women from Eastern Europe, another study noted that 50% of African-American women, who are epidemiologically at a higher risk for PPROM, had a notably low abundance of *Lactobacillus* spp. before PPROM, suggesting that bacterial composition might be particularly crucial in this group [27]. This contrasts with the non-association of *L. iners* dominance at 16 weeks with PPROM, indicating possible different etiologies for PPROM and preterm birth [37]. Additionally, women susceptible to PPROM exhibited more significant transitions between dominant bacterial species or taxa before membrane rupture, echoing recent findings of a link between decreased vaginal bacterial community stability and increased PTB risk in high-risk populations [29].

In addition to what is evident from the microbiological standpoint, there are a multitude of factors that can contribute to vaginal microflora alterations during pregnancy or potentiate the risk of preterm birth. For example, progesterone is frequently used to prevent preterm birth, particularly for women showing signs of cervical shortening during the second trimester, which is known to inhibit inflammatory pathways in reproductive tissues, reducing cytokine and prostaglandin production and potentially decreasing myometrial contractility and cervical remodeling while boosting local antimicrobial proteins [38,39]. However, progesterone’s effects might not modulate the vaginal microbiota, as evidenced by studies showing its neutral impact on the abundance of *Lactobacillus* species in women treated for a short cervix and the lack of significant microbiota alteration in non-pregnant women using hormonal contraception [40,41].

Other studies also questioned the management of cervical shortening and exposed membranes in the second trimester, noting that emergency cervical cerclage can prolong gestation and improve neonatal outcomes, but can alter the microbiota [42,43]. Intriguingly, reports found a bimodal distribution in gestational delivery age among women offered rescue cerclage, suggesting different underlying causes for cervical dilation, including subclinical infection linked to pathogen colonization and poor cerclage response [42]. This highlights the complex etiology in cases presenting with silent cervical dilatation prior to PPROM, with some women responding well to cerclage due to mechanical abnormalities and others affected by pathogenic colonization [44].

### 4.2. Study Limitations

The current study has some limitations that merit consideration. Firstly, the retrospective collection of data may lead to potential biases and inconsistencies, especially given the reliance on clinical records and digital EMR systems, which could affect the completeness and accuracy of the patient information. The exclusion criteria, while necessary for the study’s focus, limit the generalizability of the findings to all pregnant women, as those with certain conditions or treatments, such as chronic diseases or recent antibiotic use, were omitted from the analysis. Additionally, the study’s design of sampling only in the third trimester does not capture the full dynamics of the vaginal microbiome changes throughout the entire pregnancy, possibly overlooking critical early factors. The use of a convenience sample for determining the sample size might not fully represent the broader pregnant population, potentially affecting the representativeness and statistical power of the study. Lastly, the case–control design, while effective for this research, does not establish causality between the vaginal microbiota and preterm birth outcomes.

## 5. Conclusions

This research concludes that significant correlations exist between the vaginal microbiota composition and the risk of preterm birth. Elevated white blood cell and neutrophil counts increased vaginal pH, and the presence of specific bacteria, such as *Candida* spp., *Gardnerella vaginalis*, *Mycoplasma hominis*, and *Ureaplasma urealyticum*, are strongly associated with higher PTB rates. Conversely, a higher proportion of *Lactobacillus* spp. appears to confer a protective effect against PTB. These insights emphasize the potential of microbiota-focused interventions as strategies for PTB risk reduction. However, understanding the complex interplay between microbial dynamics, host immune response, and PTB is crucial for developing effective prevention and treatment methods. This study highlights the need for further comprehensive research to fully understand these relationships and develop targeted interventions to improve pregnancy outcomes.

## Figures and Tables

**Table 1:** Demographic and health characteristics of the study participants.

Variables	Preterm (n = 89)	Full-Term (n = 106)	*p*-Value *
Age (mean ± SD)	28.6 ± 4.9	29.1 ± 5.4	0.502
Age category			0.449
<35 years	62 (69.7%)	79 (74.5%)	
≥35 years	27 (30.3%)	27 (25.5%)	
BMI ≥ 30 kg/m^2^	17 (19.1%)	16 (15.1%)	0.457
Smoking during pregnancy	12 (13.5%)	8 (7.5%)	0.173
Alcohol use during pregnancy	5 (5.6%)	1 (0.9%)	0.059
Parity			0.986
Primigravida	53 (59.6%)	63 (59.4%)	
Multigravida	36 (40.4%)	43 (40.6%)	
Medical history			
UTIs	22 (24.7%)	15 (14.2%)	0.060
Hypertension	15 (16.9%)	11 (10.4%)	0.185
Diabetes	6 (6.7%)	6 (5.7%)	0.754
Anemia	16 (18.0%)	23 (21.7%)	0.517
Respiratory infections during pregnancy	6 (6.7%)	14 (13.2%)	0.138
Diarrheal illness during pregnancy	8 (9.0%)	8 (7.5%)	0.714
Others	6 (6.7%)	5 (4.7%)	0.541

*—significance threshold after Bonferroni correction = 0.0038; BMI—Body Mass Index; SD—Standard Deviation; UTI—Urinary Tract Infection.

**Table 2:** Comparative analysis of blood test results in preterm and full-term births.

Blood Tests (Median, IQR)	Preterm (n = 89)	Full-Term (n = 106)	*p*-Value *
WBCs (4.5–11.0 × 10^3^/mm^3^)	10.2 (9.7–13.1)	7.6 (5.4–9.3)	0.009
Lymphocytes (1.0–4.0 × 10^3^/mm^3^)	2.7 (2.4–3.3)	1.8 (1.2–2.9)	0.114
Neutrophils (1.5–8.0 × 10^3^/mm^3^)	7.2 (6.7–8.9)	5.1 (4.4–6.6)	<0.001
PLTs (150–450 thousands/mm^3^)	265.9 (158.0–375.9)	313.3 (269.0–425.0)	0.109
RBCs (3.9–5.5 × 10^6^/mm^3^)	4.8 (5.5–5.9)	4.4 (4.1–4.6)	0.466
Hemoglobin (12–16 g/L)	12.6 (11.5–14.0)	13.0 (12.8–14.7)	0.427
CRP (0–10 mg/L)	9.1 (4.8–12.1)	7.3 (5.5–8.0)	0.280
Creatinine (0.6–1.1 mg/dL)	0.7 (0.5–0.9)	0.8 (0.7–1.0)	0.753
Urea (7–20 mg/dL)	13.4 (10.2–16.6)	11.7 (9.5–14.8)	0.209

WBCs—white blood cells; CRP—C-Reactive Protein; RBCs—red blood cells; IQR—Interquartile Range; UTI—Urinary Tract Infection; PLTs—platelets; A *p*-value threshold of less than 0.05 was set for statistical significance; *—significance threshold after Bonferroni correction = 0.0055.

**Table 3:** Distribution of gestational characteristics and birth types.

Variables	Preterm (n = 89)	Full-Term (n = 106)	*p*-Value *
Gestational weight			<0.001
500–999 g	3 (3.4%)	0 (0.0%)	
1000–1499 g	8 (9.0%)	1 (0.9%)	
1500–2499 g	31 (34.8%)	15 (14.2%)	
>2500	47 (52.8%)	90 (84.9%)	
Gestational age			–
Early preterm (<28 weeks)	7 (7.9%)	0 (0.0%)	
Moderate preterm (28–32 weeks)	19 (21.3%)	0 (0.0%)	
Later preterm (32–36 weeks)	63 (70.8%)	0 (0.0%)	
Early term (37–38 weeks)	0 (0.0%)	18 (17.0%)	
Full-term (41–42 weeks)	0 (0.0%)	74 (69.8%)	
Post term (>42 weeks)	0 (0.0%)	14 (13.2%)	
Type of birth			<0.001
Vaginal	25 (28.1%)	78 (73.6%)	
Cesarean	58 (65.2%)	20 (18.9%)	
Assisted	6 (6.7%)	8 (7.5%)	

*—significance threshold after Bonferroni correction = 0.0166.

**Table 4:** Vaginal smear results compared between patients who gave birth preterm and full-term.

Variables	Preterm (n = 89)	Full-Term (n = 106)	*p*-Value	FDR
pH test	5.6 (5.1–6.4)	4.4 (4.0–4.7)	<0.001	0.003
Overall assessment (Nugent Score) *			0.001	0.003
Normal microbiota	35 (39.3%)	68 (64.2%)		
Intermediate microbiota	28 (31.5%)	25 (23.6%)		
Abnormal flora (bacterial vaginosis)	26 (29.2%)	13 (12.3%)		
**Particularities**				
Coccoid microbiota	17 (19.1%)	9 (8.5%)	0.029	0.043
Staphoid vaginitis	12 (13.5%)	7 (6.6%)	0.106	0.106
Fungi	21 (23.6%)	10 (9.4%)	0.007	0.014
Bacterial vaginosis and Fungi	9 (10.1%)	4 (3.8%)	0.077	0.092

*—0–3 indicates normal microbiota, 4–6 suggests intermediate microbiota, and 7–10 signifies a positive result for bacterial vaginosis; FDR—False Discovery Rate *p*-value; significance threshold after Bonferroni correction for *p*-value = 0.010.

**Table 5:** Vaginal culture results compared between patients who gave birth preterm and full-term.

Variables	Preterm (n = 89)	Full-Term (n = 106)	*p*-Value	FDR
*Actinomyces* spp.	2 (2.2%)	1 (0.9%)	0.461	0.768
*Bacillus* spp.	4 (4.5%)	3 (2.8%)	0.533	0.771
*Candida* spp.	22 (24.7%)	10 (9.4%)	0.004	0.060
*Corynebacterium* spp.	3 (3.4%)	6 (5.7%)	0.447	0.768
*Enterococcus* spp.	9 (10.1%)	12 (11.3%)	0.786	0.838
*Escherichia coli*	16 (18.0%)	10 (9.4%)	0.080	0.266
*Gardnerella vaginalis*	23 (25.8%)	13 (12.3%)	0.015	0.060
*Haemophilus influenzae*	1 (1.1%)	2 (1.9%)	0.667	0.838
*Klebsiella* spp.	7 (7.9%)	5 (4.7%)	0.362	0.750
*Lactobacillus* spp.	65 (73.0%)	93 (87.7%)	0.009	0.060
*Mycoplasma hominis*	15 (16.9%)	6 (5.7%)	0.012	0.060
*Peptostreptococcus anaerobius*	3 (3.4%)	1 (0.9%)	0.233	0.662
*Staphylococcus aureus*	5 (5.6%)	4 (3.8%)	0.540	0.771
*Staphylococcus epidermidis*	14 (15.7%)	11 (10.4%)	0.265	0.662
*Staphylococcus haemolyticus*	2 (2.2%)	3 (2.8%)	0.797	0.838
*Streptococcus agalactiae*	11 (12.4%)	9 (8.5%)	0.375	0.750
*Streptococcus anginosus*	4 (4.5%)	5 (4.7%)	0.941	0.941
*Streptococcus mitis*	2 (2.2%)	3 (2.8%)	0.797	0.838
*Streptococcus salivarius*	1 (1.1%)	2 (1.9%)	0.667	0.838
*Ureaplasma urealyticum*	13 (14.6%)	4 (3.8%)	0.007	0.060

Significance threshold after Bonferroni correction for *p*-value = 0.0020; FDR—False Discovery Rate *p*-value.

**Table 6:** Correlation matrix.

Variables (rho, *p*-Value)	Gestational Age	WBC Count	Vaginal pH	*Lactobacillus* spp.	*Candida* spp.	*Mycoplasma hominis*	*Ureaplasma urealyticum*	Nugent Score (7–10)
Gestational Age	1							
WBC Count	−0.307 *	1						
Vaginal pH	−0.452 *	0.248	1					
*Lactobacillus* spp.	0.406 *	−0.205	−0.559 *	1				
*Candida* spp.	−0.158	0.104	0.308 *	−0.256 *	1			
*Mycoplasma hominis*	−0.217 *	0.152	0.357 *	−0.312 *	0.253	1		
*Ureaplasma urealyticum*	−0.259 *	0.213	0.418 *	−0.359 *	0.158	0.504 *	1	
Nugent Score (7–10)	−0.551 *	0.324	0.672 *	−0.601 *	0.207	0.269	0.359 *	1

*—statistically significant findings at a 0.05 significance threshold; Spearman’s rho was used due to the ordinal nature of some variables, while Pearson’s correlation coefficient was calculated for continuous data.

**Table 7:** Significant risk factors associated with preterm birth.

Significant Risk Factors	Coefficient (β)	SE	OR	95% CI	*p*-Value
*Candida* spp. presence	0.61	0.27	1.84	1.1–3.0	0.018
*Gardenerella vaginalis* presence	0.83	0.31	2.29	1.3–3.9	0.003
*Lactobacillus* spp. reduction	−0.77	0.29	0.46	0.3–0.7	0.001
*Mycoplasma hominis* presence	0.68	0.26	1.97	1.2–3.2	0.007
*Ureaplasma urealyticum* presence	0.89	0.34	2.43	1.5–4.0	0.001
Vaginal pH > 4.5	0.52	0.22	1.68	1.1–2.6	0.014
Nugent Score (7–10) for bacterial vaginosis	1.04	0.38	2.83	1.7–4.7	<0.001
Fungi presence combined with abnormal microbiota	0.71	0.31	2.03	1.2–3.4	0.008

OR—odds ratio; CI—Confidence Interval; SE—Standard Error.

## Data Availability

Data are contained within the article.
